# Transfer of Carbapenem-Resistant Plasmid from *Klebsiella pneumoniae* ST258 to *Escherichia coli* in Patient

**DOI:** 10.3201/eid1606.091671

**Published:** 2010-06

**Authors:** Moran G. Goren, Yehuda Carmeli, Mitchell J. Schwaber, Inna Chmelnitsky, Vered Schechner, Shiri Navon-Venezia

**Affiliations:** Tel Aviv Sourasky Medical Center, Tel Aviv, Israel (M.G. Goren, Y. Carmeli, I. Chmelnitsky, V. Schechner, S. Navon-Venezia); National Center for Infection Control, Tel Aviv (Y. Carmeli, M.J. Schwaber, S. Navon-Venezia)

**Keywords:** Horizontal gene transfer, carbapenem-resistant plasmid, KPC, bacteria, plasmids, Klebsiella pneumoniae, Escherichia coli, antibiotic resistance, Israel, dispatch

## Abstract

*Klebsiella pneumoniae* carbapenemase (KPC) 3–producing *Escherichia coli* was isolated from a carrier of KPC-3–producing *K. pneumoniae*. The KPC-3 plasmid was identical in isolates of both species. The patient's gut flora contained a carbapenem-susceptible *E. coli* strain isogenic with the KPC-3–producing isolate, which suggests horizontal interspecies plasmid transfer.

Over the past 2 years, the extremely drug-resistant *Klebsiella pneumoniae* carbapenemase (KPC)–producing *K. pneumoniae* sequence type 258 (KpnST258) has emerged as an important nosocomial pathogen worldwide. It has spread in the United States and in various countries in Europe and Asia (*1*–*3*). The high level of antimicrobial drug resistance in this bacterium is conferred by a plasmid-encoded KPC, which confers resistance to all cephalosporins, monobactams, and carbapenems (*4*). Infection with carbapenem-resistant *K. pneumoniae* is associated with an increased proportion of deaths compared to carbapenem-susceptible *K. pneumoniae* (*5*). Although *Klebsiella* with plasmid-mediated carbapenem resistance is a major risk to hospitalized patients, spread of these resistance plasmids into *Escherichia coli* poses an even greater public health threat because resistant *E. coli* may become part of the normal gut flora and thereby become a notable source of infections among sick and the healthy persons in healthcare settings and in the community (*6*).

In 2008, a carbapenem-nonsusceptible *E. coli*–producing KPC-3 isolate (Eco2) was identified in Tel Aviv Sourasky Medical Center in Israel. Until this case, carbapenem resistance in *E. coli* at the hospital was related exclusively to KPC-2 production (*7*). KPC production in *E. coli* remains rare worldwide, even in areas where KPC-producing *K. pneumoniae* isolates are identified. We aimed to investigate the origin of KPC-3 in this *E. coli* isolate and to explore a possible molecular and epidemiologic link between the presence of *bla*_KPC-3_ in this species and in the KpnST258 strain prevalent in our hospital.

## The Study

In April 2008 a carbapenem-nonsusceptible *E. coli* strain, marked as Eco2, was recovered from the gall bladder drainage of a 91-year-old man with dialysis-dependent end-stage renal disease, congestive heart failure, anemia, and peptic ulcer disease. A month earlier, the patient had been hospitalized with sepsis that developed after an infected heel wound had required amputation of the left leg below the knee. The patient was treated with ertapenem, metronidazole, colistin, and vancomycin. Acute cholecystitis developed, and the patient underwent cholecystostomy. During his hospital stay, the patient underwent screening for carriage of carbapenem-resistant *Enterobacteriacae* (CRE) as part of a routine infection control program aimed at limiting the spread of CRE. Two rectal swabs were collected 1 week apart. The first swab specimen was negative for CRE by culture, and the second swab specimen showed a carbapenem-resistant *K. pneumoniae* strain (marked Kpn1), which was PCR positive for *bla*_KPC_. One month after the patient’s admission, a carbapenem-nonsusceptible *E. coli* (Eco2) was isolated from drainage at the cholecystectomy site, which prompted this study.

Microbiologic and molecular investigations (pulsed-field gel electrophoresis [PFGE], DNA isolation, isoelectric focusing analysis [IEF], PCR detection of resistance genes, plasmid isolation, transformation, and Southern analysis) were performed as described (*2**,**4**,**7*). The carbapenem-nonsusceptible *E. coli* isolated from the clinical specimen (Eco2) was initially identified by Vitek-2 **(**bioMérieux, Marcy-l’Etoile, France) as resistant to imipenem (MIC>16 mg/L). Further antimicrobial-drug susceptibility testing of the strain by using agar dilution and Etest (AB Biodisk, Solna, Sweden) showed MICs in the resistant range for ceftriaxone and aztreonam; in the intermediate range for ceftazidime and piperacillin/tazobactam; and in the susceptible range for cefepime, ertapenem, meropenem, imipenem, aminoglycosides, quinolones, tigecycline, and colistin (Appendix Table). IEF identified 2 β-lactamases with isoelectric pH values of 5.4 and 6.7, corresponding to those of TEM-type and KPC. β-lactamase gene PCR screening and sequencing indicated the presence of *bla*_TEM-1_ and *bla*_KPC-3_. Results of screening for other β-lactamase genes were negative.

**Figure 1:**
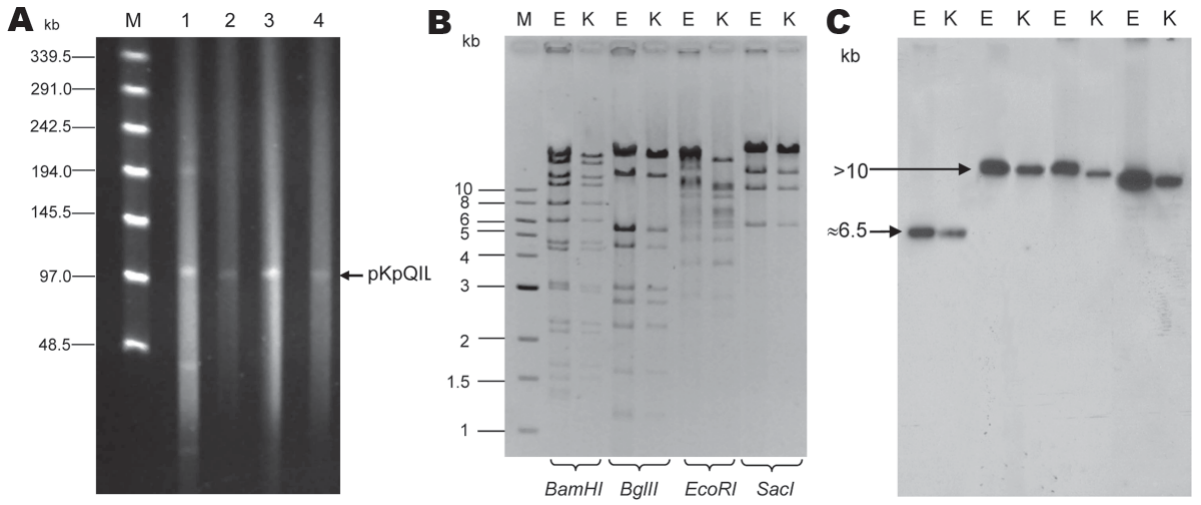
A) Analysis of *Klebsiella pneumoniae* carbapenemase (KPC)–encoding plasmids in isolates Kpn1 (1), Eco2 (3), Kpn1-T (2), and Eco2-T (4), Israel, 2008. Plasmid size estimation was performed by digestion of DNA with S1 nuclease (20 U; Promega, Madison, WI, USA) followed by pulsed-field gel electrophoresis (PFGE) with the CHEF-DR III apparatus (Bio-Rad Laboratories, Inc., Hercules, CA, USA), as described (*8*–*11*). Lambda ladder PFG marker (New England Biolabs, Beverly, MA, USA) was used as a molecular size marker (lane M). B) Restriction fragment length polymorphism of the KPC-3–encoding plasmid from Kpn1-T (K) and Eco2-T (E). Plasmid DNA was digested with *Bam*HI, *Bgl*II, *Eco*RI, and *Sac*I endonucleases (New England Biolabs) and underwent PFGE on a 1% agarose gel. The level of similarity between restriction patterns was calculated by using GelcomparII software version 5 (Applied Maths, Kortrigk, Belgium). Lane 1, 1-kb DNA ladder (New England Biolabs). C) Southern blot analysis of plasmid DNA hybridized with *bla*_KPC-3_-labeled probe. Plasmid restriction products were transferred to a Hybond N^+^ membrane (Amersham Biosciences, Little Chalfont, United Kingdom), cross-linked with UV light, and hybridized with a *bla*_KPC-3_-labeled probe (892-bp product of *bla*_KPC-3_).

**Figure 2:**
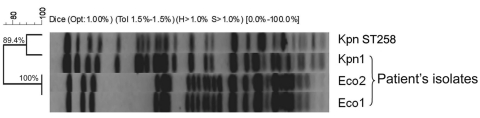
Pulsed-field gel electrophoresis demonstrating genetic relatedness of study isolates Eco2, Eco1, and Kpn1, and a representative *Klebsiella pneumoniae* isolate of the epidemic clone, Kpn ST258, Israel, 2008. Bacterial DNA was prepared and cleaved with 20U *Spe*I endonuclease (New England Biolabs, Beverly, MA, USA), followed by electrophoresis in a CHEF-DR III apparatus (Bio-Rad Laboratories, Inc., Hercules, CA, USA), as described (*4*). The macrorestriction patterns of the isolates were compared according to the Dice similarity index (1.5% tolerance interval) by using GelcomparII software (Applied Maths, Kortrigk, Belgium).

Transformation of plasmids purified from Eco2 into an *E. coli* DH10B recipient strain (Eco2-T) indicated transfer of a single plasmid that encoded these *bla*_TEM-1_ and *bla*_KPC-3_ (Figure 1, panel A), and increased the MICs of the recipient strains to broad-spectrum cephalosporins and carbapenems (Appendix Table). PFGE identified the genetic similarity between the colonizing *Klebsiella* (Kpn1) and a representative KpnST258 (isolate Kpn557 described previously [*4*]) (Figure 2). Susceptibility testing of Kpn1 reflected the extremely drug-resistant phenotype characteristic of isolates belonging to this clone (*12*) (Appendix Table).

We compared plasmids of Kpn1 and Eco2. Kpn1 carried 4 different plasmids, whereas Eco2 carried 1 plasmid that correlated with the 105-kb plasmid of Kpn1. Experiments to transform Eco2 and Kpn1 plasmids into an *E. coli* DH10B recipient, followed by selection on plates containing 100 µg/mL ampicillin and screening for *bla*_KPC_-positive colonies, showed that DH10B was transformed with the105-kb KPC-3–encoding plasmid (Figure 1, panel A). This plasmid correlated in size with that of pKpQIL, the KPC-3-encoding plasmid of *Klebsiella* ST258 in Israel (*13*). Plasmid DNA restriction fragment length polymorphism showed that band patterns of the 2 KPC-3–encoding plasmids of Kpn1 and Eco2 were highly similar (98% similarity) (Figure 1, panel B), and Southern analysis with a *bla*_KPC-3_ probe showed the same hybridization pattern (Figure 1, panel C).

We aimed to determine whether the patient’s gut was colonized with a carbapenem-susceptible *E. coli* strain, which would ultimately serve as the in vivo recipient of the *bla*_KPC-3-_encoding plasmid. Thus, the first broth culture prepared (obtained before the patient was colonized with KPC-3–producing *E. coli*) was processed. Aliquots (0.1 mL) were directly plated onto a MacConkey agar plate (Hy-Labs, Rehovot, Israel). *E. coli* colonies isolated from the plate were restreaked onto a MacConkey agar plate, yielding an *E. coli* strain 7364 (Eco1) that was susceptible to all antimicrobial drugs tested (Appendix Table). PFGE DNA fingerprinting showed that Eco1 was 100% identical to the KPC-3-producing clinical strain Eco2, isolated from the clinical specimen (Figure 2). Plasmid analysis of this strain, however, proved that it lacked plasmid pKpQIL (results not shown).

Elements belonging to KPC transposon Tn*4401*, including *tnpA, tnpR, ISKpn6,* and *ISKpn7* (*14*), were identified by PCR and sequencing on both KPC-3–encoding plasmids originating from Kpn1 and Eco2. These genetic determinants were absent in the susceptible Eco1. These data suggest that Eco1 has acquired pKpQIL from Kpn1 in the patient’s gut, leading to the formation of Eco2. Although acquisition of the plasmid increased MICs for imipenem, meropenem, and ertapenem considerably, it did not confer full resistance (Appendix Table) presumably due to copy number of the plasmid or the expression level of *bla*_KPC-3_ in *E. coli*. Curing of pKpQIL from Eco2 was performed by sequential transfers at an elevated temperature (42°C). The cured strain, which lacked the KPC-encoding plasmid, showed full susceptibility to all antimicrobial drugs tested, similar to the Eco1 strain isolated from the patient’s gut flora.

The patient received a combination of 4 antimicrobial agents concomitantly (ertapenem, metronidazole, colistin, and vancomycin) during the period in which Eco1 acquired in vivo the plasmid pKpQIL, thereby becoming Eco2. We believe that the selection pressure imposed by these antimicrobial agents contributed to the sequence of events that led to plasmid transfer. We hypothesize that interspecies conjugation and antimicrobial pressure led to the preferential selection of Eco2, rather than Eco1, as a determinant of infection in this patient.

Interspecies KPC transfer can presumably occur through the dissemination of mobile genetic elements as has been described for transfer of the *mec*A gene between strains of *Staphylococcus aureus* (*15*). *bla*_KPC_ may spread through transfer by virtue of its location on the Tn*4401* transposon (*14*), or by dissemination of the intact KPC-encoding plasmid, likely through natural conjugation. Multiple attempts to mimic the natural transfer of pKpQIL, the KPC-3-encoding plasmid from Kpn1 into Eco1 by using conjugation experiments were not successful. The isolation of an isogenic, antimicrobial drug–susceptible *E. coli* clone enabled us to decipher the natural order of the interspecies genetic transfer event

## Conclusions

With increasing global spread of KPC-producing *K. pneumoniae* ST258, the likelihood increases of interspecies transfer of drug-resistance determinants into a highly fit *E. coli* clone. Such an event may have severe public health consequences, leading to elimination of any effective antimicrobial drug treatment against the most common human bacterial pathogens.

## Supplementary Material

Appendix Table Patient's bacterial isolates and Escherichia coli DH10B transformed with their KPC-3-encoding plasmids, Israel, 2008*
