# Supplementary material for: Transfer of Carbapenem-Resistant Plasmid from Klebsiella pneumoniae ST258 to Escherichia coli in Patient
Source: Emerg Infect Dis. 2010 Jun;16(6):1014–7. doi: 10.3201/eid1606.091671 (PMC3086234; doi:10.3201/eid1606.091671)
Supplement: Appendix Table — Patient's bacterial isolates and Escherichia coli DH10B transformed with their KPC-3-encoding plasmids, Israel, 2008* [file 09-1671-appT-s1.pdf]

Appendix Table. Patient's bacterial isolates and *Escherichia coli* DH10B transformed with their KPC-3–encoding plasmids, Israel, 2008*

Bacteria	Date of isolation	Source	KPC-3 β-lactamase	MIC (mg/L)†													
				CTX	CAZ	FEP	AZT	TZP	AMK	GEN	CIP	LFX	IMI‡	MER‡	ETP‡	TCG§	COL§
Eco2	2008 Apr	Gall bladder fluid	+	>64	16	2	>64	64	4	<1	<0.25	<0.25	2	0.38	2	0.125	0.094
Eco2-T¶	–	Laboratory strain	+	8	>64	2	>64	64	<2	<1	<0.25	<0.25	<0.5	0.38	2	0.125	0.047
Kpn1	2008 Mar	Rectal swab	+	>64	>64	>64	>64	>128	>64	4	>4	>8	16	>32	>32	1	0.047
Kpn1-T#	–	Laboratory strain	+	8	16	<1	>64	64	<2	<2	<0.25	<0.25	0.5	0.38	1	0.125	0.19
Eco1	2008 Mar	Rectal swab	–	<1	<1	<1	<1	<4	<2	<1	<0.25	<0.25	0.19	0.012	0.006	0.19	0.047
E. coli DH10B**	–	Laboratory strain	–	<0.25	<0.25	0.125	<1	<1	<1	<4	<2	<1	<0.5	0.25	0.023	0.125	0.047

*KPC, *Klebsiella pneumoniae* carbapenemase; CTX, ceftriaxone; CAZ, ceftazidime; FEP, cefepime; AZT, aztreonam; TZP, piperacillin tazobactam; AMK, amikacin; GEN, gentamicin; CIP, ciprofloxacin; LFX, levofloxacin; IMI, imipenem; MER, meropenem; ETP, ertapenem; TGC, tigecycline; COL, colistin.
†Susceptibility testing was performed by Vitek-2 (bioMérieux, Marcy-l'Etoile, France), unless stated otherwise.
‡MICs of carbapenems were tested by agar dilution. MICs <0.5 mg/L were determined by Etest (AB Biodisk, Solna, Sweden).
§MICs of colistin and tigecycline were determined by Etest.
¶Eco2-T that acquired the KPC-3-encoding plasmid from Eco2.
#Kpn1-T that acquired the KPC-3-encoding plasmid from Kpn1.
**The recipient *E. coli* strain used in the transformation experiments.
